# Guidance for Administering Biologics for Severe Asthma and Allergic Conditions

**DOI:** 10.1155/2022/9355606

**Published:** 2022-09-10

**Authors:** Delbert R. Dorscheid, Jason K. Lee, Warren Ramesh, Mark Greenwald, Jaime Del Carpio

**Affiliations:** ^1^Division of Respiratory and Critical Care Medicine, St. Paul's Hospital, Center for Heart Lung Innovation, University of British Columbia, Vancouver, British Columbia V6Z 1Y6, Canada; ^2^Clinical Immunology & Allergy Internal Medicine, Evidence Based Medical Educator Inc. & Urticaria Canada, Biologics and Therapeutics Committee, ACAAI, Toronto M5G 1E2 ON, Canada; ^3^Royal Alexandra Hospital, Edmonton Respiratory Consultants, Edmonton, Alberta T5J 3S9, Canada; ^4^University of Toronto and Queen's University, Toronto, Ontario M5S 1A1, Canada; ^5^Division of Allergy and Clinical Immunology, McGill University, Montreal H3A 0G4, Quebec, Canada

## Abstract

Asthma is a common respiratory disorder in Canada for which biologics may be prescribed for poorly controlled illness. Treatment with biologics, however, is sometimes inappropriately discontinued due to misconceptions regarding their potential immunologic effects, and concerns surrounding their continued use in severe asthma during the COVID-19 pandemic continue to propagate. Biologics can still be administered in a majority of health and treatment conditions. With regard to cardiac-related issues such as hypertension or cardiovascular disease (CVD), there is no solid evidence that suggests biologics should be withheld, as the benefits of treatment outweigh the risks. Asthmatic patients on biologic treatment should also continue treatment if they have, or are currently being treated for, a respiratory infection, including COVID-19. Evidence also indicates the importance of maintaining asthma control to reduce the risk of severe COVID-19 infection. Biologic treatment can be administered in severe asthmatic patients with bronchiectasis, though further evidence is needed to better understand the benefits. Biologic treatment should be continued postsurgery to reduce postoperative respiratory complications, as well as throughout the course of pregnancy. Regarding concerns over vaccine administration, nearly all vaccines can be given without interruption of biologic treatment in patients with severe asthma or allergic conditions. Appropriate screening for respiratory illnesses, such as COVID-19, continues to be warranted in clinical practices to reduce the risk of transmission. As recommendations from public health and regulatory agencies have been lacking, this guidance document addresses the administration of biologics in different health circumstances and respiratory illness screening during the COVID-19 pandemic.

## 1. Introduction

Asthma affects approximately 11% (3.8 million children and adults) of the Canadian population [1]. Approximately 5% to 10% of patients have severe asthma, which is defined by the Canadian Thoracic Society and similarly by Global Initiative for Asthma (GINA) guidelines as asthma requiring a high-dose steroid inhaler (i.e., QVAR® RediHaler® (beclomethasone dipropionate HFA), Pulmicort® Turbuhaler® (budesonide dry powder for oral inhalation), Alvesco® (ciclesonide), Flovent (fluticasone propionate inhalation aerosol/power), Arnuity® Ellipta® (fluticasone furoate inhalation powder), or Asmanex® Twisthaler® (mometasone furoate inhalation powder)), and a second controller or oral steroids for 6 months or longer to keep asthma controlled [2, 3]. Allergic conditions, including chronic spontaneous urticaria, chronic rhinosinusitis with nasal polyps, atopic dermatitis, and eosinophilic granulomatosis with polyangiitis, among others, may occur with or without asthma and can be difficult to treat successfully; patients may ultimately require surgical intervention. A number of biologic treatments, which include Xolair® (omalizumab), Fasenra® (benralizumab), Nucala (mepolizumab), Dupixent® (dupilumab), and Cinqair® (reslizumab), are available for patients with poorly controlled asthma, eosinophilic asthma, or allergic conditions [4–9]. Early and rapid diagnosis of severe asthma phenotypes, such as eosinophilic asthma, through the use of point-of-care assessment of biomarkers (e.g., blood eosinophil count), could accelerate the time needed to define severe asthma phenotypes and help predict the most suitable biologic treatment for patients with severe eosinophilic phenotypes [10].

These treatments target specific factors in the immune response seen in severe type 2 inflammatory asthma and other allergic conditions and have been shown to provide safe and efficacious control of the disease. However, in daily practice, biologic treatment (e.g., administration of a dose) is sometimes withheld, delayed, or stopped inappropriately because of misperceptions of potential immunologic effects of biologic treatments in patients presenting with other illnesses or who have recently or are scheduled to receive vaccinations. For example, in an analysis of a Canadian prescription claims database (IQVIA), approximately 50% and 60% of patients discontinued omalizumab and mepolizumab, respectively, 2 years after starting treatment [11, 12]. In addition, many physicians have reported that their patients have missed a dose of asthma or allergic biologic treatment, potentially due to unfounded concerns.

The biologic treatments used in severe asthma and allergic conditions are considered immunomodulators, which are treatments that increase or decrease immune response by targeting specific molecules [13]. Immunomodulatory treatments differ from immunosuppressants (e.g., steroids) because they act selectively on a target molecule in the immune system rather than suppressing the immune system overall [13, 14]. The biologics affect different primary targeted components of the immune response seen in severe asthma and allergic conditions and also have indirect effects on the immune system. The primary targets of the biologic treatments are as follows [4–9]:

  Omalizumab: immunoglobulin E (IgE) and the IgE receptor
  Reslizumab and mepolizumab: interleukin (IL)-5
  Benralizumab: IL-5 receptor
  Dupilumab: IL-4 and IL-13 through the IL-4 receptor

The biologic treatments listed above are initiated in a clinic setting, and all except for omalizumab and reslizumab (which are always administered at a clinic) can be continued in the clinic or administered at home. These treatments are critical for achieving and maintaining disease control and improving patient health and quality of life.

Evidence of the beneficial effects of biologic treatments in severe asthma have been shown in clinical and real-world studies. Several phase II and III clinical trials have demonstrated the efficacy of reslizumab in reducing blood eosinophil counts, improving pulmonary function and asthma control, and reducing the rate of exacerbations [15]. In a multicenter, retrospective study in patients with severe asthma, treatment with dupilumab reduced the number of exacerbations and oral corticosteroid use while improving pulmonary function and asthma control [16]. Similar effectiveness was observed with mepolizumab in a single-center, retrospective study in patients with severe eosinophilic asthma with or without comorbidities (e.g., nasal polyps, allergic rhinitis, and bronchiectasis) [17]. Treatment with benralizumab also showed clinical and functional improvements in a patient with severe allergic eosinophilic asthma and relapsing nasal polyps [18]. As such, it is imperative that patients remain in therapy with as few treatment interruptions as possible and that healthcare providers and clinicians understand the benefits of administering biologic treatment and the risks of withholding it. During the COVID-19 pandemic, this issue has become more evident because of the scenarios that healthcare providers face on a daily basis, from patients presenting with symptoms of illness, testing positive for COVID-19, or being hospitalized with symptoms to determining when biologic treatment may be administered in relation to the receipt of the COVID-19 vaccine. Given that there is little guidance on these scenarios from public health and regulatory agencies, the objectives of this document are to provide general guidance for Canadian healthcare providers regarding the appropriate administration of biologic treatments and to provide recommendations for screening of respiratory illness for patients coming into the clinic for their biologic treatment, with a focus on COVID-19 infection. A summary document of the guidance detailed below is included in the supplementary material.

## 2. Results and Discussion, Section 1: When to Withhold Biologic Treatment

Sometimes, when a patient arrives at the clinic to receive the next dose of their biologic treatment, the dose is withheld due to concern for a patient's condition, such as a comorbid respiratory illness, or fear of the increased risk of adverse events due to recent administration of a vaccine or antibiotics, or plan for upcoming surgery. However, in clinical expert opinion, there are very few situations in which the biologic treatment for severe asthma and allergic conditions should be withheld.  Table 1 summarizes conditions where there may be a concern for administration; the checkmarks designate that the biologic treatment can be given. Specific situations where withholding the dose of biologic may be considered are detailed further in this section.

### 2.1. Cardiac-Related Issues (Hypertension and Chronic Chest Pain)

There is a concern related to comorbid cardiovascular disease (CVD) and the use of biologics for asthma/allergic conditions, as some biologics may increase the risk of a cardiovascular event, as seen in other inflammatory diseases. Studies that have assessed CVD risk with biologics have shown mixed results [19–21].

Increased IgE levels have been seen in some acute CVD events (i.e., myocardial infarction (MI) and acute coronary syndrome) and in patients with a history of CVD [22, 23], which may be of concern with omalizumab because of its anti-IgE effects. In a 5-year, postmarketing, observational study of patients with severe asthma (EXCELS study), more patients receiving omalizumab had cardiovascular events, including transient ischemic attack (TIA), MI, pulmonary hypertension, pulmonary thrombosis, and unstable angina compared to patients not receiving omalizumab (13.4 patients per 1,000 patient-years vs. 8.1 patients per 1,000 patient-years, respectively) [24]. However, several characteristics of the study, such as the nonrandomized design and the fact that the study was not designed specifically to assess the incidence of cardiovascular events, higher CVD risk at baseline for patients receiving omalizumab, and the small absolute number of cardiovascular events, make it difficult to determine the true risk of cardiovascular events in patients receiving omalizumab [24]. Neither the Canadian Thoracic Society nor GINA recommends holding omalizumab for CVD [2, 3]; based on the guidelines and expert opinion, omalizumab can be given to patients with a history of CVD.

There is some evidence suggesting that IL-5 may have protective effects against the development of arterial plaque [25], but there is currently no evidence that IL-5 inhibitors (mepolizumab, reslizumab, and benralizumab) increase the risk of CVD. No studies have evaluated dupilumab and the risk of CVD or hypertension.

The presence of chronic chest pain may be a concern for healthcare providers because it may be symptomatic of a more severe type of asthma in which the airway is occluded by mucus, causing cough and chest pain, as well as decreased lung delivery of aerosol medications [26]. However, there is no evidence to support that the dose of biologic for severe asthma or allergic condition should be withheld in patients with chronic chest pain.

In summary, no solid evidence has shown that biologic treatment should be withheld for hypertension or CVD. Documentation of hypertension or chest pain at the time of administration may be of value. Because the benefits of biologic treatment outweigh the risks, biologic administration for severe asthma or allergic conditions is recommended regardless of cardiac-related issues.

### 2.2. Infection (Signs or Symptoms of Infection or Antibiotic Use)

As biologics are immunomodulatory and not immunosuppressive, no data have shown that the mechanism of action of any of the biologics increases the susceptibility or seriousness of infection, including COVID-19. This section reviews the signs and symptoms of respiratory infection, use of antibiotics, and parasitic infections as related to the administration of biologics in severe asthma and allergic conditions.

#### 2.2.1. Respiratory Infections

Although respiratory infections and rhinosinusitis were reported as adverse events in the clinical trials with benralizumab, mepolizumab, and omalizumab, the incidence was low and comparable to placebo; there are no precautions or warnings with the use of benralizumab or mepolizumab with respiratory illnesses in the prescribing information [4–6, 8, 9]. With omalizumab and reslizumab, viral infections and upper respiratory tract infections were some of the most commonly observed adverse events, but they occurred at similar rates in patients receiving active treatment or placebo [4, 9]. No study has shown that signs or symptoms of infection, including fever or recent antibiotic use, are a reason for withholding the dose of the biologic. Therefore, based on clinical expert opinion, biologic treatment for severe asthma or allergic conditions should be administered regardless of respiratory symptoms.

#### 2.2.2. Parasitic Infections

Although not common in Canada, parasitic helminth infections may be a reason to withhold biologic treatment. Helminths include ascariasis, tapeworm (*Echinococcosis*), hookworm, and whipworm, which can present with no symptoms in a mild infection to severe gastrointestinal symptoms and growth retardation in children in a severe infection [27]. Patients who are receiving any biologic for severe asthma or allergic conditions should receive treatment for the parasitic infection before starting the biologic [4–6, 8, 9]. If the helminth infection occurs during ongoing biologic treatment and the patient is not responding to the helminth treatment, the biologic should be discontinued until the infection resolves.

#### 2.2.3. COVID-19 Infection

With the COVID-19 pandemic, there has been an additional concern with the continuation of treatment with biologics. The concern is twofold: A) Should all patients continue to receive their biologic treatment during the COVID-19 pandemic? B) If a patient tests positive for COVID-19, should the dose of biologic still be administered during active infection? The following is in response to those specific questions:

- Biologic treatments should be continued throughout the COVID-19 pandemic. The Canadian Thoracic Society, United States (US) Centers for Disease Control and Prevention (CDC), and GINA recommended the continuation of biologic treatment, as withholding treatment in severe asthma could increase the risk of severe exacerbations seen with viral infection [28]. Guidelines from the US and Europe provide similar recommendations [29]. Additionally, a retrospective cohort study of asthmatic patients who underwent polymerase chain reaction (PCR) testing for severe acute respiratory syndrome coronavirus 2 (SARS-CoV-2) demonstrated that biologic use was not associated with an increased risk of contracting COVID-19 [30]. Thus, given the known benefits of continuing biologics and the risk of exacerbations from discontinuing their use, patients should continue to receive their biologics for severe asthma and allergic conditions during the COVID-19 pandemic.
- For patients who have tested positive for COVID-19, the biologic dose should still be administered. The concern around administration is related to infection control and preventing the spread of COVID-19 during treatment administration rather than the risk of COVID-19 affecting the efficacy or safety of the biologic. Patients who are hospitalized with COVID-19 can still receive their biologic, assuming stringent infection control is in place. The same retrospective cohort study mentioned in the previous response also showed that biologics were not associated with a significantly increased risk of worse COVID-19 outcomes [30]. See Section 2 for guidance on when patients with COVID-19 infection may return to the clinic.

Asthmatic patients have been found to be less likely to contract COVID-19 or require hospitalization once infected. A recent systematic literature review and meta-analysis of 57 studies demonstrated a 14% risk reduction in acquiring COVID-19 (*P* < 0.0001) and a 13% reduction in hospitalization from COVID-19 (*P*=0.03) for asthmatic patients compared to nonasthmatic patients [31]. Another recent systematic literature review and meta-analysis of 131 studies did not identify an association between asthma and a higher risk of intubation or mechanical ventilation once hospitalized (relative risk (RR): 1.03; 95% confidence interval (CI): 0.72, 1.46), and patients with asthma were shown to have a lower risk of death compared to patients without asthma (RR: 0.65; 95% CI: 0.45, 0.98) [32].

The evidence still suggests the importance of maintaining asthma control, especially during the COVID-19 pandemic, as patients taking asthma medications have lower odds of severe COVID-19 outcomes, including hospitalization and intensive care unit admission, than those not taking medication [33]. According to GINA guidance, asthmatic patients with well-controlled, mild-to-moderate asthma do not have an increased risk of severe COVID-19 [34]. However, patients with severe asthma are shown to have a significantly increased risk of COVID-related mortality compared to patients on asthma therapy [35]. Patients with recent clinical visits for asthma care are at increased risk for severe COVID-19 outcomes, suggesting that patients with uncontrolled asthma could be vulnerable to more serious infection [33].

In summary, patients should continue to receive biologics for severe asthma and allergic conditions, even if they have a current infection, including COVID-19, and signs or symptoms of illness or are receiving antibiotic treatment. Patients should also maintain control of asthma symptoms through appropriate therapy to reduce the risk of severe COVID-19 illness.

### 2.3. Bronchiectasis

Bronchiectasis is one of the more common asthma-associated, heterogeneous comorbidities that increases airway inflammation, leading to higher exacerbation rates and impairment of respiratory function [36]. The coexistence of type 2 (T2) high severe asthma (T2-SA) and bronchiectasis has recently been considered as an emerging phenotype of severe and difficult-to-treat asthma that leads to a worsening prognosis of the disease [37]. The pathogenesis of bronchiectasis can be attributed to long-term eosinophil-mediated inflammatory damage, which is promoted by T2 inflammation and induces tissue changes and airway remodeling [37].

The efficacy of biologics targeting T2 or IL-5 inflammation in patients with severe asthma and bronchiectasis has been evaluated in a case series and a pilot study [37–39]. Results from a case series of 12 patients with bronchiectasis who were treated with anti-T2 biologics, including dupilumab and omalizumab, demonstrated effectiveness in reducing monthly respiratory exacerbations, systemic corticosteroid courses, and systemic antibiotic courses [38]. In a pilot study evaluating mepolizumab, an anti-IL-5 agent, in 4 patients with severe, uncontrolled asthma with bronchiectasis, significant improvement was observed in pulmonary function, as well as a significant reduction in exacerbations and in blood and sputum eosinophils after 1 year of treatment [39]. As such, biologics can potentially provide clinical benefit in patients with severe asthma with comorbid bronchiectasis, though further larger studies are needed to determine their role in this patient population. In regard to when to withhold biologic treatment, similar reasoning applies in patients with severe asthma without bronchiectasis.

### 2.4. Surgery

The risk of postoperative infection or complication may be increased with the use of immunomodulatory biologics [40]. While studies evaluating this risk have been conducted, most have been in other disease states (e.g., rheumatoid arthritis and ulcerative colitis) or with biologics with different mechanisms of action, such as tumor necrosis factor-*α* inhibitors (e.g., infliximab and etanercept) [40, 41]. There is currently no evidence to support the concern that biologics used in severe asthma and allergic conditions increase the risk of postsurgical complications; as such, on the basis of clinical expert opinion, the dose of the biologics for severe asthma and allergic conditions does not need to be withheld in preparation for surgery or postsurgery and will more likely reduce postoperative respiratory complications.

### 2.5. Pregnancy

In Canada, the prevalence of asthma ranges from 10% to 17% in women of childbearing age (aged 15 to 49 years) [1]. As with nearly all medications, the fetal risk is the greatest concern. However, disease exacerbations are often more common during pregnancy, and poorly controlled asthma can increase the risk of preterm delivery, low birth weight, and preeclampsia [3]. For example, in a population-based Swedish registry study of 33,829 pregnancies in mothers with asthma, exacerbations were associated with 1.45 times the risk of low birth weight, as well as 1.50 times and 1.35 times the risk of elective and emergency cesarean section, respectively [42]. Because the benefits of treatment (i.e., prevention of exacerbations) outweigh the risks, it is generally recommended that patients continue asthma medications, including biologics, during pregnancy [3].

Benralizumab, mepolizumab, reslizumab, and dupilumab have not been studied in pregnant women, although benralizumab and mepolizumab have ongoing pregnancy registries [5, 6, 8]. The EXPECT study, an observational study of 250 pregnant women who used omalizumab during pregnancy, showed no evidence of increased risk of congenital abnormalities, prematurity, low birth weight, or babies small for gestational age [43, 44]. If a patient is newly pregnant and has not started a biologic, it is suggested to wait until they are no longer pregnant before beginning biologic treatment. Otherwise, biologic treatment should be continued through the course of the pregnancy.

### 2.6. Vaccinations

Vaccination is particularly beneficial in patients with chronic diseases, and many immunizations prevent or decrease symptoms of respiratory illnesses, such as influenza and pneumonia. Vaccine technology continues to evolve, and many types of vaccines are now used, including inactivated vaccines, live-attenuated vaccines, messenger ribonucleic acid (mRNA) vaccines, conjugate vaccines, toxoid, and viral vector vaccines [45]. Inactivated, mRNA, conjugate, toxoid, and viral vector vaccines contain organisms or parts of organisms that are not able to cause disease but provide immunity to infection. Live-attenuated vaccines are weakened versions of a pathogenic virus or bacterium. There has been concern that if a vaccine is administered with current biologic treatment, the immunomodulatory effects of biologics in combination with the immune response triggered by the vaccine could exacerbate disease or put patients at greater risk for vaccine-related adverse events [46]. Little evidence exists, however, that patients receiving biologics and any vaccine are at risk for disease exacerbation or adverse events. Guidance for each vaccine type and whether it can be administered with current biologic treatment is shown in Table 2 (the checkmark indicates that the biologic can be safely administered with the vaccine).

Overall, patients with severe asthma and allergic conditions can receive vaccinations without interruption to biologic treatment. A multidisciplinary committee of gastroenterologists, dermatologists, rheumatologists, and infectious disease specialists with expertise in vaccinology met recently to develop recommendations on the use of vaccinations in patients using immunosuppressive treatments, based on the GRADE (Grading of Recommendation, Assessment, Development, and Evaluation) criteria [46]. The guidance is as follows:

  If patients have not yet started a biologic treatment, vaccine status should be assessed, and any vaccine that is needed should be given before initiating biologic treatment [46].
  For patients who are currently receiving biologic treatment, inactivated vaccines, which includes mRNA, conjugate, toxoid, and viral vector vaccines, can be administered without issue. For the optimal response to the vaccine, it is suggested that the vaccine be given based on the half-life of the biologic; for example, in a patient receiving mepolizumab every 4 weeks, a vaccine could be given approximately 2 or 3 weeks after administration of mepolizumab, as the half-life of mepolizumab is 16 to 22 days [8, 46].
  For live vaccines, data have demonstrated that mepolizumab, omalizumab, and benralizumab can be continued. However, live vaccines should be avoided while receiving treatment with dupilumab and reslizumab [4, 5]. Guidelines suggest that when the benefit of a live vaccine outweighs the risks, biologic treatment should be interrupted based on the half-life of the biologic and can be resumed once the viremia of the vaccine is cleared (e.g., the duration of viremia for the varicella vaccine is 14 days) [46]. For dupilumab, if a live vaccine is to be administered, dupilumab should be held for 1 month or the vaccine should be given 2 weeks before a patient starts dupilumab.

The new technologies used in the COVID-19 vaccines have raised questions regarding the safety of their use in patients receiving biologics. The COVID-19 vaccines available in Canada include the mRNA vaccines by Pfizer-BioNTech and Moderna and the viral vector vaccines by Johnson & Johnson, Oxford-Astra-Zeneca, and Verity Pharmaceuticals-Serum Institute of India. Although the mRNA and viral vector vaccines work slightly differently, it is important to recognize that none of them are live virus vaccines, and all can be given safely to patients receiving biologic treatment for allergy or asthma [34, 47, 48]. The Canadian Thoracic Society stated specifically, “*There is no biological rationale as to why anti-IgE (omalizumab), anti-IL5 (reslizumab and mepolizumab), anti-IL5R (benralizumab) or even anti-IL4/13 (dupilumab) treatments should place patients at higher risk for adverse events* [47].”

Additionally, there has been some concern with the mRNA vaccines and the risk of myocarditis and pericarditis, particularly in males ages 16 years and older who have received the vaccine [49, 50]. Patients may present with acute chest pain and shortness of breath [49]. If these symptoms are new and the patient received the COVID-19 vaccine recently, additional tests such as an electrocardiogram or C-reactive protein may be prudent. However, there is no evidence to suggest that patients with severe asthma or allergic conditions or patients receiving biologics for severe asthma or allergic conditions are at greater risk for myocarditis or pericarditis [49, 50].

Therefore, COVID-19 vaccines should be administered to all patients on biologic treatment, and the dose of biologic should not be withheld while receiving the vaccination. The Canadian Thoracic Society, GINA, and US CDC all suggest not administering the vaccine and biologic on the same day and if possible, to vaccinate between doses of the biologic, although this is based on the presumption of safety and not on any clinical evidence [34, 47, 48].

In summary, any inactivated vaccine, including COVID-19 vaccines, can be given without interruption of biologic treatment in patients with severe asthma or allergic conditions. Live vaccines can be given with all biologic treatments except dupilumab and reslizumab; if the vaccine benefits outweigh the risks, dupilumab and reslizumab can be held temporarily for the administration of the live vaccine.

## 3. Results and Discussion—Section 2: Screening for Respiratory Illness in Patients Coming into the Clinic to Receive Treatment

Screening for respiratory illnesses, such as influenza and COVID-19, has been incorporated into clinical practice for quite some time. The objective of screening for respiratory illness is to assess the infectious risk posed by the ill patient to other patients, visitors, and staff at the healthcare site rather than to determine whether a patient should receive biologic treatment [51]. As stated above, biologic treatment should be given, even to patients who are ill. The logistics regarding whether an ill patient should come to the clinic or when a patient is no longer considered infectious, however, may dictate the timing of the biologic dose. Screening for respiratory illness should include questions about whether the patient has had the following symptoms [52]:

- New onset or worsening of existing cough
- Presence of fever (>38°C)
- New onset of headache, sore throat, joint pain, muscle pain, or severe fatigue

If a patient has had a) or/and b) and 1 symptom in c), further assessment is needed, and the patient may need to wait to come into the clinic until the infection resolves or be placed in a separate room from other patients for infection control.

### 3.1. COVID-19

A similar process should be used for screening for COVID-19 [53]. Patients should also be asked if they are feeling sick differently than their usual symptoms of asthma or allergic conditions. Patients should be asked about additional symptoms of COVID-19, such as new loss of smell or taste, gastrointestinal symptoms, runny nose, congestion, conjunctivitis, and shortness of breath, as well as the symptoms mentioned for influenza. Temperature checks should be continued, and a rapid test should be done if available, although there is a risk of false positives.

Caution is warranted for COVID-19-positive patients entering the clinic, as the infection could be spread to other patients and staff. Some experts have suggested the adoption of the “10/20/40 rule” for when patients with COVID-19 infection can return to the clinic. Recommendations, albeit subjective, for coming into the clinic are based on the severity of the COVID-19 infection:

  For patients who have a mild infection or were asymptomatic, patients should wait a minimum of 10 days until after the positive test before entering the clinic [54].
  For patients hospitalized with COVID-19 symptoms, the recommendation is to wait a minimum of 20 days until after the positive test [54].
  For patients who required mechanical ventilation or had additional complications, the expert clinical opinion suggests waiting 40 days before coming into the clinic.

## 4. Conclusions

In conclusion, biologics used in severe asthma and allergic conditions can be given in the majority of circumstances. Control of asthma is essential for improving patient outcomes and preventing exacerbations; as such, there are few situations where withholding the dose of the biologic outweighs the benefits of good asthma control. As more data and clinical evidence become available, updates can be made to this guidance document [55, 56].

## Figures and Tables

**Table 1:** When can biologic treatment be administered?.

	Biologic				
	Xolair (omalizumab)	Fasenra (benralizumab)	Nucala (mepolizumab)	Dupixent (dupilumab)	Cinqair (reslizumab)
Patient's condition, treatment/vaccine, or timing					
Hypertension	✔	✔	✔	✔	✔
Fever	✔	✔	✔	✔	✔
Chronic chest pain	✔	✔	✔	✔	✔
Pneumonia or other respiratory illness	✔	✔	✔	✔	✔
Antibiotics	✔	✔	✔	✔	✔
Active parasitic (helminth) infection	Hold until treatment is completed	Do not give	Do not give	Do not give	Do not give
Before or after surgery	✔	✔	✔	✔	✔
Headache	✔	✔	✔	✔	✔
Pregnancy^a^ and breastfeeding	✔	✔	✔	✔	✔
Inactivated vaccine	✔	✔	✔	✔^b^	✔
Live-attenuated vaccines	✔	✔	✔	Do not give	Do not give

^a^Biologics should not be initiated during pregnancy, but current treatment should be continued. ^b^Some physicians may consider holding treatment based on a risk/benefit discussion of optimal immunity.

**Table 2:** Types of vaccines and whether to administer with a biologic [4, 5, 6, 8, 9, 45].

Vaccine type	Examples of available vaccines^a^	OK to receive a vaccine with continued biologic use?
Inactivated	Influenza, hepatitis A, rabies	✔
Live-attenuated	MMR, rotavirus, varicella	✔
		Except dupixent and cinqair^b^
mRNA	Pfizer-BioNTech COVID-19, Moderna COVID-19	✔
Conjugate, subunit, recombinant, polysaccharide	Hepatitis B, HPV, pneumococcal, meningococcal, shingles	✔
Toxoid	Diphtheria, tetanus	✔
Viral vector	Johnson & Johnson COVID-19, Oxford-AstraZeneca COVID-19, Verity Pharmaceuticals-Serum Institute of India COVID-19	✔

HPV: human papillomavirus; MMR: measles, mumps, and rubella; mRNA: messenger ribonucleic acid. ^a^Table is not comprehensive; review all vaccine product information before administering. ^b^Dupixent and Cinqair doses should be held for 1 month before the live vaccine administration and reinitiated at least 2 weeks postvaccination.

## Data Availability

No data were used to support the development of this position paper.

## Supplementary Materials

Supplementary Materials The following items were uploaded in a separate file, per the author guidelines of *Canadian Respiratory Journal*: Guidance for Administering Biologics for Severe Asthma and Allergic Conditions: Summary Document. Supplementary Table 3; When is it ok to administer a biologic? Supplementary Table 4; Types of vaccines and whether to administer with a biologic.
