# Supplementary material for: Guidance for Administering Biologics for Severe Asthma and Allergic Conditions
Source: Can Respir J. 2022 Sep 10;2022:9355606. doi: 10.1155/2022/9355606 (PMC9482537; doi:10.1155/2022/9355606)
Supplement: Supplementary Materials — The following items were uploaded in a separate file, per the author guidelines of Canadian Respiratory Journal: Guidance for Administering Biologics for Severe Asthma and Allergic Conditions: Summary Document. Supplementary Table 3; When is it ok to administer a biologic? Supplementary Table 4; Types of vaccines and whether to administer with a biologic. [file 9355606.f1.docx]

**Supplementary Material**

**Guidance for Administering Biologics for Severe Asthma and Allergic Conditions: Summary Document**

**Question (Q): Are there certain circumstances (eg, infection, hypertension, recent vaccine use) where a biologic should not be given to patients with severe asthma and allergic conditions?**

**Answer (A):** In the majority of circumstances, the dose of the biologic can be given (see Supplementary Table 3). Adequate control of severe asthma and allergic conditions is essential, particularly when there is risk of other respiratory diseases. Refer to Supplementary Table 3 for recommendations based on expert opinion.

**Supplementary Table 3. When is it ok to administer a biologic?**

|  | **Biologic** | | | | |
| --- | --- | --- | --- | --- | --- |
|  | **Xolair (omalizumab)** | **Fasenra (benralizumab)** | **Nucala (mepolizumab)** | **Dupixent (dupilumab)** | **Cinqair (reslizumab)** |
| **Patient’s condition or treatment** | | | | | |
| Hypertension | 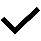 | 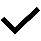 | 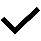 | 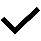 | 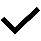 |
| Fever | 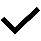 | 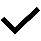 | 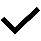 | 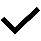 | 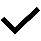 |
| Chronic chest pain | 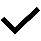 | 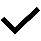 | 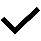 | 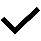 | 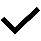 |
| Pneumonia or other respiratory illness | 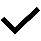 | 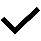 | 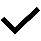 | 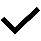 | 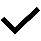 |
| Antibiotics | 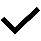 | 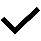 | 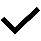 | 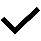 | 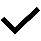 |
| Active parasitic (helminth) infection | **Hold dose until treatment is completed** | **Do not give** | **Do not give** | **Do not give** | **Do not give** |
| Before or after surgery | 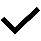 | 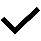 | 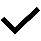 | 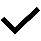 | 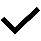 |
| Headache | 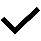 | 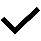 | 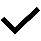 | 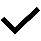 | 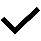 |
| Pregnancy^a^ and breastfeeding | 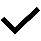 | 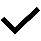 | 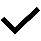 | 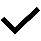 | 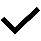 |
| Inactivated vaccine | 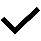 | 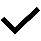 | 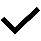 | 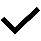^b^ | 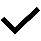 |
| Live-attenuated vaccine | 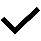 | 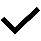 | 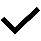 | **Do not give** | **Do not give** |

^a^ Biologics should not be initiated during pregnancy, but current treatment should be continued.

^b^ Some physicians may consider withholding treatment based on a risk/benefit discussion of optimal immunity.

**Q: Are there certain vaccines that should be avoided with biologic treatment? What about the COVID-19 vaccines in particular?**

**A:** Nearly all vaccines can be given when a patient is receiving biologic treatment (see Supplementary Table 4). Caution is warranted with Dupixent or Cinqair use and administration of live vaccines. If a live vaccine is required and the patient is on Dupixent or Cinqair, the dose should be held for 1 month before the vaccine administration and reinitiated at least 2 weeks post-vaccination.

None of the COVID-19 vaccines are live, and as such, can be safely administered to patients receiving biologics for severe asthma or allergic conditions. Patients receiving biologics for severe asthma or allergic conditions are at no greater risk than the normal population for myocarditis or pericarditis after receiving the mRNA vaccines, so any of the COVID-19 vaccines can be used. If possible, administration of the COVID-19 vaccine and the biologic should not occur on the same day, and the vaccine should be given between doses of the biologic, although there is no clinical evidence to confirm this as a requirement.

**Supplementary Table 4. Types of vaccines and whether to administer with a biologic**

| **Vaccine type** | **Examples of available vaccines^a^** | **Ok to receive with biologic use?** |
| --- | --- | --- |
| Inactivated | Influenza, hepatitis A, rabies | 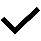 |
| Live-attenuated | MMR, rotavirus, varicella | 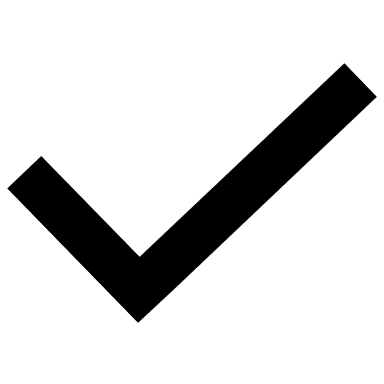  **Except Dupixent and Cinqair^b^** |
| mRNA | Pfizer-BioNTech COVID-19, Moderna COVID-19 | 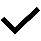 |
| Conjugate, subunit, recombinant, polysaccharide | Hepatitis B, HPV, pneumococcal, meningococcal, shingles | 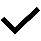 |
| Toxoid | Diphtheria, tetanus | 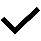 |
| Viral vector | Johnson & Johnson COVID-19, Oxford-AstraZeneca COVID-19, Verity Pharmaceuticals-Serum Institute of India COVID-19 | 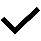 |

Abbreviations: HPV – human papillomavirus; MMR – measles, mumps, and rubella; mRNA – messenger RNA.

^a^ Table is not comprehensive; review all vaccine product information before administering.

^b^ Dupixent and Cinqair doses should be held for 1 month before the live vaccine administration and reinitiated at least 2 weeks post-vaccination.

**Q: Should we continue to screen for respiratory illness and COVID-19 when patients arrive at the clinic?**

**A:** Yes, as this is one of the best ways to prevent the spread of respiratory illnesses. Temperature and assessment of symptoms for influenza and COVID-19 should be done upon patient’s arrival at the clinic, regardless of the patient’s COVID-19 vaccination status. If the patient recently tested positive for COVID-19 or has positive symptoms for a respiratory illness, the patient should be sent home and rescheduled for their dose of biologic once no longer infectious (following the 10/20/40 rule for COVID-19) or should be isolated in a room from other patients to receive the biologic dose if rescheduling is not possible or benefits of treatment outweigh the risk. Ideally, as rapid point-of-care testing for COVID-19 becomes more widely available, all patients entering the clinic should receive a rapid COVID-19 test, particularly if patients are unvaccinated or not fully vaccinated. However, with the currently available testing modalities, for patients who are vaccinated, asymptomatic, and have had no recent exposures to COVID-19, the US CDC and the Provincial Infectious Disease Advisory Committee are not recommending COVID-19 testing [55, 56].
